# Impact of protein supplementation during endurance training on changes in skeletal muscle transcriptome

**DOI:** 10.1186/s12864-020-6686-x

**Published:** 2020-06-09

**Authors:** Pim Knuiman, Roland Hangelbroek, Mark Boekschoten, Maria Hopman, Marco Mensink

**Affiliations:** 1 Division of Human Nutrition, Wageningen University & Research, Stippeneng 4, 6708 WE Wageningen, The Netherlands; 2 School of Biomedical Sciences, University of Leeds, Clarendon Way, Leeds, LS2 9JT UK; 3 Advanced Analytics, Viqtor Davis B.V., Parijsboulevard 143 A, 3541 CS Utrecht, The Netherlands; 4 Department of Physiology, Radboud University Medical Centre, Geert Grooteplein-West 32, 6525 GA Nijmegen, The Netherlands

## Abstract

**Background:**

Protein supplementation improves physiological adaptations to endurance training, but the impact on adaptive changes in the skeletal muscle transcriptome remains elusive. The present analysis was executed to determine the impact of protein supplementation on changes in the skeletal muscle transcriptome following 5-weeks of endurance training.

**Results:**

Skeletal muscle tissue samples from the *vastus lateralis* were taken before and after 5-weeks of endurance training to assess changes in the skeletal muscle transcriptome. One hundred and 63 genes were differentially expressed after 5-weeks of endurance training in both groups **(**q-value< 0.05). In addition, the number of genes differentially expressed was higher in the protein group (PRO) (892, q-value< 0.05) when compared with the control group (CON) (440, q-value< 0.05), with no time-by-treatment interaction effect (q-value> 0.05). Endurance training primarily affected expression levels of genes related to extracellular matrix and these changes tended to be greater in PRO than in CON.

**Conclusions:**

Protein supplementation subtly impacts endurance training-induced changes in the skeletal muscle transcriptome. In addition, our transcriptomic analysis revealed that the extracellular matrix may be an important factor for skeletal muscle adaptation in response to endurance training. This trial was registered at clinicaltrials.gov as NCT03462381, March 12, 2018.

**Trial registration:**

This trial was registered at clinicaltrials.gov as NCT03462381.

## Background

Skeletal muscle is an extraordinary malleable tissue which is demonstrated by its rapid remodeling and adaptation to exercise training [1, 2]. Repetitive bouts of endurance exercise, e.g. endurance training, lead to various metabolic and morphological adaptations in skeletal muscle [3, 4]. At the myocellular level, long term skeletal muscle adaptation is supposed to be the result of repeated modifications in transcriptional and translational responses of each exercise bout thereby increasing the synthesis of specific proteins required for remodeling [5–8]. However, training-induced changes in baseline transcriptome have also shown to play an important role [9–11]. Skeletal muscle transcriptome analysis provides an unbiased examination of the molecular alterations to exercise training, thereby potentially unravelling novel pathways involved in adaption to endurance training [12–14].

Protein feeding following endurance exercise has shown to affect mRNA-specific pathways involved in extracellular matrix, myogenesis, immunogenic response, and energy metabolism [15], suggesting that repeated post-exercise endurance protein feeding may enhance the adaptive response to endurance training. Whether protein supplementation also impacts the changes in the skeletal muscle transcriptome following a period of endurance training remains to be elucidated. We have recently demonstrated that protein supplementation during endurance training enhances physiological adaptations, where the major part of the adaptations was observed during the first 5-weeks of the 10-weeks training intervention [16]. Therefore, we decided to specifically focus the present analysis on the effect of protein supplementation on changes in skeletal muscle transcriptome during 5-weeks of endurance training. To this end, we assessed the impact of protein supplementation during 5-weeks of endurance training on changes in the skeletal muscle transcriptome. We hypothesize that protein supplementation elicits greater changes in the skeletal muscle transcriptome when compared to carbohydrate supplementation.

## Results

### Baseline characteristics

In total, four subjects dropped out during the conduction of the study for various reasons. Analysis was executed on the 40 subjects who completed the 5-weeks training program (CON: *n* = 21 vs PRO: *n* = 19). Baseline characteristics were not different between groups and can be found in Table 1.

**Table 1:** Baseline characteristics and physiological effects of 5-weeks endurance training. Values are means ± standard deviation. P-values are from mixed model analysis. CON = control group. PRO = protein group

	CON group	(*n* = 21)	PRO group	(*n* = 19)	*P-values*		
	0 weeks	5 weeks	0 weeks	5 weeks	Training	Treatment	Interaction
Age (yr)	22.5 ± 2.3		21.5 ± 1.6				
Body mass (kg)	77.2 ± 7.2		76.3 ± 5.4				
Height (m)	1.85 ± 0.1		1.85 ± 0.1				
BMI (kg/m^-2^)	22.4 ± 1.3		22.3 ± 1.5				
Lean mass (kg)	61.0 ± 4.2	61.1 ± 4.1	60.1 ± 4.8	61.6 ± 5.3	= 0.0001	= 0.9	= 0.000
Fat mass (kg)	12.8 ± 4.5	12.7 ± 4.6	12.8 ± 2.9	12.2 ± 3.1	= 0.02	= 0.8	= 0.089
VO_2max_ (L·min^-^^1^)	3.9 ± 0.3	4.1 ± 0.3	3.8 ± 0.4	4.2 ± 0.5	= < 0.0001	= 0.7	= 0.004
VO_2max_ (mL·kg^-^1·min^-^1)	50.8 ± 3.9	53.0 ± 4.9	49.9 ± 3.4	54.9 ± 4.8	= < 0.0001	= 0.7	= 0.016
Citrate synthase (μmol·g ^−^ 1·min ^−^ 1)	21.8 ± 5.4	28.7 ± 4.4	23.4 ± 6.2	31.9 ± 5.2	= < 0.0001	= 0.1	= 0.206
Time-trial performance (seconds)	982.3 ± 86.1	871.1 ± 45.8	957.8 ± 106.5	839.1 ± 53.4	= < 0.0001	= 0.1	= 0.796

### Endurance training program and effect

For more detailed information regarding the endurance training program and supplementation strategy the reader is referred to our recently published paper [16]. Briefly, the monitored training sessions were performed between 0900 and 2100. Exercise training adherence, intensity and supplementation adherence were not different between groups, a . Five weeks of endurance training significantly increased maximal aerobic capacity and skeletal muscle oxidative capacity. Protein supplementation caused a greater gain in maximal aerobic capacity and stimulated lean mass accretion but did not further increase skeletal muscle oxidative capacity and endurance performance (Table 1). A full discussion of the physiological effects of endurance training with or without protein supplementation can be found elsewhere [16].

### Muscle transcriptome

Endurance training differentially expressed gene in the muscle transcriptome in both the CON and the PRO group. The activity of more genes was altered by endurance training in the PRO group than in the CON group (893 vs. 441, respectively, F-test q-value < 0.05). Table 2 shows the top 20 significant genes based on level of significance for both CON group and PRO group. Among the top 20 significant genes for the CON group are genes related to extracellular matrix organization including collagen type IV alpha chain (COL4A2), collagen type IV alpha 1 chain (COL4A1), laminin subunit alpha 4 (LAMA4), laminin subunit beta 1 (LAMB1) and alpha-2-macroglobulin (A2M). Top 20 significant genes for the PRO group were comparable with those of the CON group and relate to extracellular matrix organization including collagen type III alpha 1 chain (COL3A1), secreted protein acidic and cysteine rich (SPARC), collagen type IV alpha 2 chain (COL4A2), collagen type IV alpha 1 chain (COL4A1), laminin subunit alpha 4 (LAMA4), peroxidasin (PXDN), laminin subunit beta 1 (LAMB1), alpha-2-macroglbulin (A2M) and nidogen 1 (NID1).

**Table 2:** Top 20 significant genes in the CON and PRO group sorted on level of significance (F-test q-value< 0.0001) in the CON (**A**) and PRO (**B**) group. Q-values for CON and PRO group as well as the interaction effect of endurance exercise training with protein supplementation are adjusted IMBT *p*-values. FC is the signed fold change. CON is the change in the control group. PRO is the change in the protein group. Inter is the interaction effect between protein supplementation and endurance training

(A). Gene	FC CON	FC PRO	Q-val. CON	Q-val. PRO	P-val. Inter	Q-val. Inter
*LAMA4*	1.39	1.57	0.000	0.000	0.051	0.821
*COL4A1*	1.69	1.87	0.000	0.000	0.311	0.933
*A2M*	1.22	1.33	0.000	0.000	0.029	0.796
*MYO1B*	1.37	1.44	0.000	0.000	0.476	0.957
*CD34*	1.28	1.33	0.000	0.000	0.496	0.958
*NFIX*	−1.12	−1.09	0.000	0.001	0.288	0.926
*THBS4*	1.61	1.94	0.000	0.000	0.081	0.853
*COL4A2*	1.54	1.77	0.000	0.000	0.144	0.881
*RYR3*	1.42	1.12	0.000	0.336	0.003	0.647
*FXYD1*	−1.15	−1.13	0.000	0.000	0.612	0.974
*COX4I1*	1.19	1.15	0.000	0.001	0.441	0.952
*TMEM159*	−1.41	−1.23	0.000	0.026	0.083	0.854
*SMTNL1*	−1.55	− 1.39	0.000	0.003	0.298	0.929
*LAMB1*	1.46	1.79	0.000	0.000	0.025	0.782
*ALDH1B1*	1.35	1.25	0.000	0.005	0.254	0.916
*RHOJ*	1.32	1.15	0.000	0.090	0.035	0.799
*SMOC2*	1.31	1.37	0.000	0.000	0.482	0.958
*LXN*	1.41	1.28	0.000	0.007	0.254	0.916
*ANKRD29*	1.42	1.16	0.000	0.198	0.020	0.756
*DECR1*	1.19	1.17	0.000	0.000	0.767	0.989
(**B). Gene**	**FC PRO**	**FC CON**	**Q-val. CON**	**Q-val. PRO**	**P-val. Inter**	**Q-val. Inter**
*LAMA4*	1.57	1.39	0.000	0.000	0.051	0.821
*A2M*	1.33	1.22	0.000	0.000	0.029	0.796
*LAMB1*	1.79	1.46	0.000	0.000	0.025	0.782
*COL4A1*	1.87	1.69	0.000	0.000	0.311	0.933
*THBS4*	1.94	1.61	0.000	0.000	0.081	0.853
*COL4A2*	1.77	1.54	0.000	0.000	0.144	0.881
*MYO1B*	1.44	1.37	0.000	0.000	0.476	0.957
*NID1*	1.48	1.26	0.002	0.000	0.022	0.756
*CD34*	1.33	1.28	0.000	0.000	0.496	0.958
*SPARC*	1.45	1.29	0.000	0.000	0.075	0.848
*COL15A1*	1.43	1.25	0.001	0.000	0.045	0.816
*UTRN*	1.22	1.12	0.003	0.000	0.029	0.796
*EDNRB*	1.70	1.34	0.005	0.000	0.016	0.756
*PXDN*	1.75	1.48	0.000	0.000	0.107	0.863
*ETS1*	1.43	1.26	0.001	0.000	0.063	0.839
*MXRA5*	2.51	1.90	0.000	0.000	0.110	0.863
*COL3A1*	2.06	1.70	0.000	0.000	0.178	0.893
*IGFBP7*	1.35	1.26	0.000	0.000	0.233	0.914
*ANXA5*	1.35	1.10	0.268	0.000	0.001	0.558
*CAPN6*	1.83	1.46	0.003	0.000	0.060	0.836

### Effect of protein supplementation

**Fig. 1:**
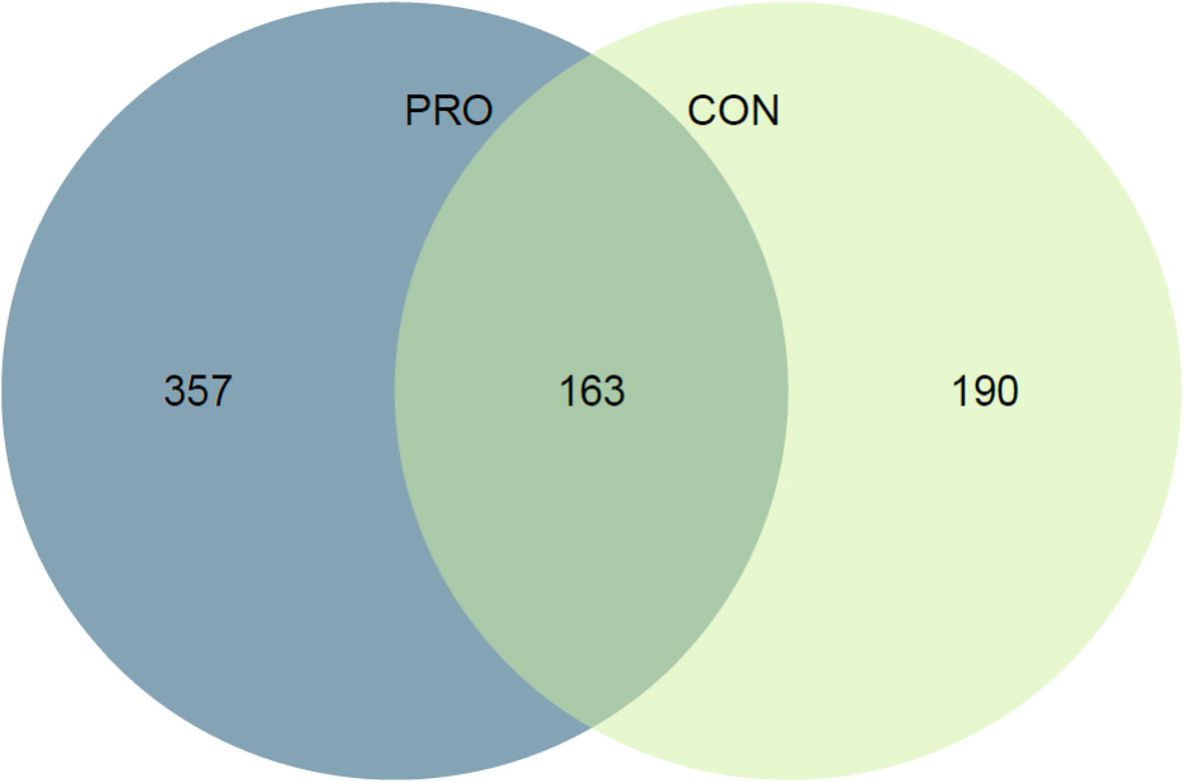
Venn diagram showing the number of differentially expressed genes per group. Selected genes (F-test q-value< 0.05) for each the CON group and the PRO group and the groups combined (raw *p*-value< 0.0001)

**Fig. 2:**
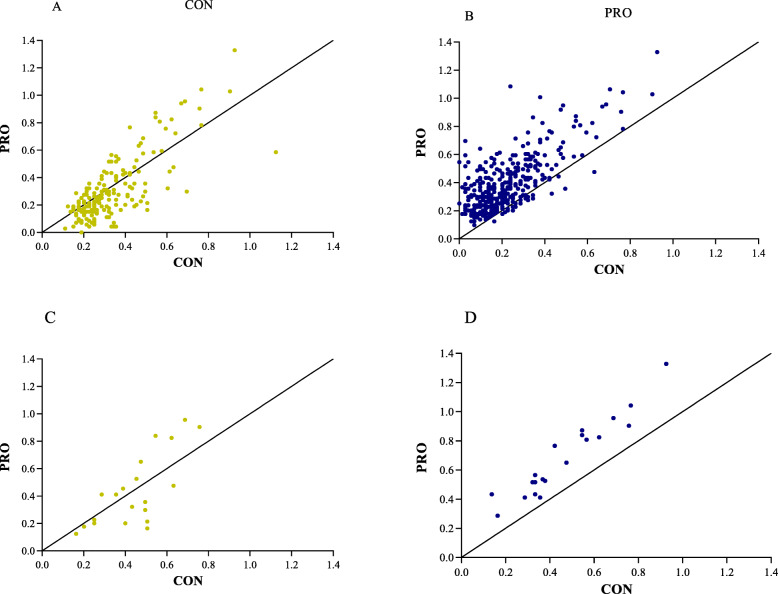
Scatterplots with line of identity to visualize the magnitude of change in muscle transcriptome per group. Figs. A & B are based on the total number of genes changed per group (184 for CON (**a**) and 384 for PRO (**b**), F-test q-value< 0.05). Figs. C & D are based on the top 20 significant genes changes in the CON (**c**) and PRO (**d**) group

**Fig. 3:**
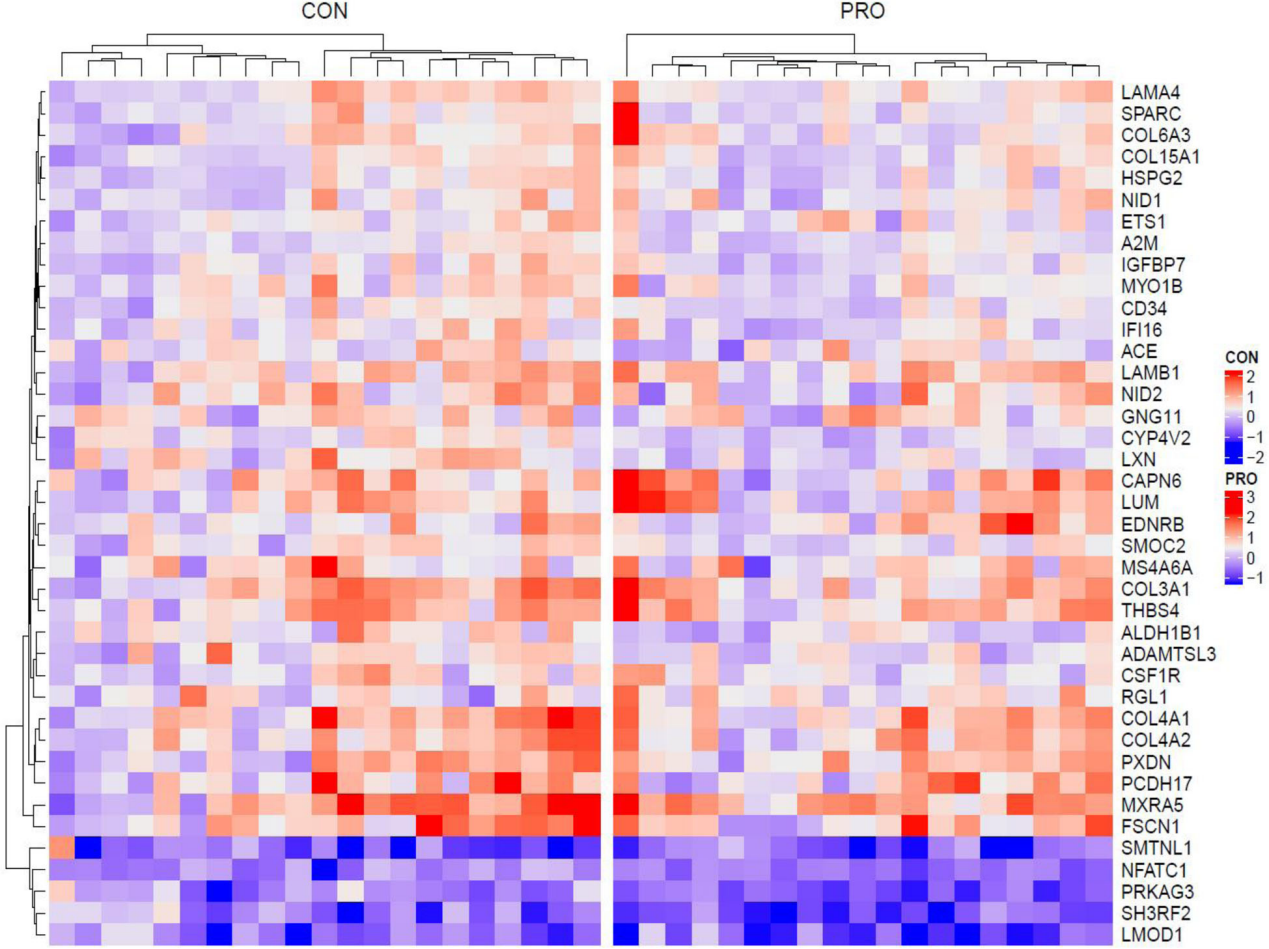
Heatmap of changes in gene expression per group. (F-test q-value< 0.0001) in the CON (left) and PRO (right) groups

Figure 1 (Venn diagram) shows the number of genes regulated as a result of endurance training for each the CON group and the PRO group and the groups combined. After 5-weeks of endurance training, gene expression count was greater in the PRO group compared with CON. In addition, the top 20 and overall gene transcript change in muscle transcriptome was consistently greater in the PRO group when compared to the CON group (Fig. 2). Figure 3 shows a heatmap of the genes that were differentially expressed by endurance training in both the CON group and PRO group (40 genes, F-test q-value< 0.0001). The changes in gene expression following 5-weeks of endurance training did not markedly differ between the CON group and PRO group (time-by treatment interaction, F-test q-value> 0.05). No major differences can be observed with regard to training response between the PRO and CON group. Gene-set-enrichment analysis showed a similar result, as gene sets that were significant for the CON group were generally also significant for the PRO group.

### Biological processes

Based on all significant genes altered (F-test q-value< 0.05) in each group, gene ontology biological processes revealed extracellular matrix organization as the process with the highest change in gene expression profile in both the CON group and the PRO group (Table 3). In the CON group 25 genes were linked to extracellular matrix organization whereas 55 genes in the PRO group. Accordingly, gene set enrichment analysis (Table 4) showed time-by treatment interaction for extracellular matrix organization processes such as extracellular matrix receptor interaction (q-value< 0.001), extracellular matrix glycoproteins (q-value = 0.006) and collagen formation (q-value = 0.041). Gene set enrichment also showed significant increases in energy metabolism and oxidative phosphorylation with no clear differences between the CON group and the PRO group (q-value> 0.05).

**Table 3:** Top 10 gene ontology biological processes from EnrichR regulated in the CON group (**A**) and PRO group (**B**) based on the total number of genes that was significantly regulated in the CON group (*n* = 440) and PRO group (*n* = 892) group (F-test q-value< 0.05)

A	Name of biological process	Genes (*n*)	P-value	Q-value
1	extracellular matrix organization	25	0.000	0.000
2	sarcomere organization	7	0.000	0.002
3	muscle contraction	19	0.000	0.000
4	positive regulation of sprouting angiogenesis	7	0.000	0.001
5	positive regulation of B cell differentiation	3	0.001	0.041
6	regulation of angiogenesis	16	0.000	0.001
7	mitochondrial ATP synthesis coupled proton transport	3	0.012	0.180
8	regulation of release of sequestered calcium ion into cytosol	8	0.000	0.003
9	actomyosin structure organization	11	0.000	0.000
10	myofibril assembly	8	0.000	0.002
**B**	**Name of biological process**	**Genes (*n*)**	**P-value**	**Q-value**
1	extracellular matrix organization	55	0.000	0.000
2	regulation of smooth muscle cell migration	8	0.001	0.000
3	mitochondrial ATP synthesis coupled proton transport	5	0.002	0.075
4	collagen fibril organization	11	0.000	0.000
5	regulation of angiogenesis	25	0.000	0.000
6	positive regulation of cell migration	27	0.000	0.001
7	positive regulation of smooth muscle cell migration	4	0.001	0.049
8	regulated exocytosis	19	0.000	0.005
9	basement membrane organization	4	0.000	0.026
10	cellular protein modification process	76	0.000	0.001

**Table 4:** Top 10 significant enriched gene sets in both the CON group and the PRO group (interaction effect). CON is the training, Inter is the interaction effect. ES is the enrichment score. The ES reflects the degree to which the genes in a gene set are overrepresented at the top or bottom of the entire ranked list of genes

GSEA	Name of biological process	ES CON	ES Inter	q-value CON	q-value Inter
1	Kegg ECM receptor interaction	0.70	0.58	0.000	0.000
2	Naba core matrisome	0.73	0.47	0.000	0.000
3	Naba ECM glycoproteins	0.73	0.46	0.000	0.006
4	Pid integrin1 pathway	0.69	0.54	0.000	0.007
5	Reactome integrin cell surface interactions	0.63	0.51	0.000	0.012
6	Biocarta RHO pathway	0.81	0.62	0.000	0.019
7	Pid TCR pathway	0.64	0.51	0.000	0.032
8	Reactome collagen formation	0.60	0.52	0.000	0.041
9	Pid syndecan 1 pathway	0.73	0.53	0.000	0.051
10	Pid integrin 3 pathway	0.53	0.54	0.000	0.051

## Discussion

We have recently demonstrated that protein supplementation enhances physiological adaptations to endurance training. The greater physiological adaptations elicited by protein supplementation were mainly observed during the first 5 weeks of training of a 10 week endurance training intervention [16]. Likewise, changes in the skeletal muscle transcriptome were primarily observed during the first 5 weeks of training with no further changes from week 5 to 10 weeks of training. Therefore, to gain further insight regarding the effects of protein supplementation during endurance training on changes in the skeletal muscle transcriptome, the present analysis focused on changes in skeletal muscle transcriptome during 5 weeks of endurance training.

Five weeks of endurance training increased maximal aerobic capacity. Adding protein supplementation elicited a greater increase in maximal aerobic capacity and stimulated lean mass gain. For a more detailed discussion on the changes in physiological outcome measures the reader is referred to our recently published paper [16]. At the skeletal muscle transcriptional level, endurance training caused relatively small (FC < 2) but consistent and statistically robust changes in the skeletal muscle transcriptome. Furthermore, changes in the skeletal muscle transcriptome tended to be greater in the PRO group as compared to the CON group. However, the differences in changes in the skeletal muscle transcriptome between the two groups are far less clear. This lack of clear differences in skeletal muscle gene expression transcripts between the PRO and CON group is likely due to timing of muscle tissue sampling, low sample size and high inter-individual variation.

In this study we demonstrated that the physiological adaptive response to endurance training was accompanied by significant changes in the skeletal muscle transcriptome. Gene set enrichment analysis showed that endurance training caused significant changes in gene expression transcripts involved in extracellular matrix, which is in line with previous reports that have investigated changes in skeletal muscle transcriptome following prolonged endurance training [13, 14]. Several upregulated genes among the top 20 genes are involved in extracellular matrix organization, including COL4A2, COL4A1, LAMA41, LAMB1 and A2M. The results of gene-ontology biological processes and gene set enrichment analysis are consistent with the top 20 genes, showing increased extracellular matrix remodeling. The observed changes in gene expressions transcripts related to extracellular matrix remodeling tended to be more pronounced in the PRO group than the CON group. The latter suggests that the greater changes in skeletal muscle transcriptome, in particular the extracellular matrix, may reflect the greater physiological adaptations observed in the PRO group (e.g. greater gain in VO_2max_ and stimulation of lean mass accretion).

The extracellular matrix is composed of collagen, glycoproteins and proteoglycans [17]. Moreover, extracellular matrix remodeling is a primary adaptation to endurance training [4]. The extracellular matrix is important for muscle cell development, structure maintenance, force transmission, and tissue remodeling through the modulation of growth factors and extracellular molecule interactions [18]. Extracellular matrix degradation is an important morphological adaptation by allowing growth of new capillaries from existing ones in response to endurance training [19–24]. Whether the exercise-induced growth of capillaries was further stimulated by protein supplementation and contributed to the larger increase in maximal aerobic capacity cannot be concluded from these data.

Our observation that protein supplementation may increases extracellular matrix remodeling to endurance training is new and further elaborates on previous work, which demonstrates that addition of protein to post-exercise carbohydrate-lipid nutrition differentially alters the transcriptome involved in tissue structure and remodeling through regulation of extracellular matrix [15]. General skeletal muscle adaptations to exercise training include regulation of angiogenesis, mitochondrial biogenesis, myogenesis and alterations in structural support such as the extracellular matrix [25, 26]. There is surprisingly little known about the role of the extracellular matrix in response to endurance training. Our data show that the gene expression transcriptional response to endurance training in skeletal muscle is related to extracellular matrix components and that protein supplementation tended to enlarge this adaptive response. In this study, it could be that the extent in which the extracellular matrix remodeled reflects the degree of muscle growth. Lean mass substantially increased in the protein group and this was accompanied by stronger regulations in gene expression transcripts related to extracellular matrix remodeling. Previous research postulated that remodeling of the extracellular matrix is required for exercise-training induced muscle growth [27].

In contrast to the observed effect of protein supplementation on physiological adaptation, we were unable to find a clear additional effect of protein supplementation on the skeletal muscle transcriptome besides the extracellular matrix. It is possible that the effects of protein supplementation already started to manifest during the early hours of recovery from exercise, when mRNA abundance generally peaks [8, 28]. Although the precise mechanisms by which protein supplementation elicited a greater increase in maximal aerobic capacity to endurance training cannot be derived from this analysis, it is likely that protein supplementation enhanced the gene/protein expression changes after each exercise session thereby improving skeletal muscle tissue adaptation, resulting in cumulatively meaningful changes in recovery and phenotypic adaptation over a prolonged period of time.

## Conclusion

Thus far, much attention has been given to the acute molecular responses to a single bout of exercise, and the current theory suggests that acute signals predict/drive phenotypic adaptation over time. For example, the AMP-activated protein kinase and peroxisome proliferator-activated receptor-y coactivator-1ɑ, have been proposed as primary regulators of muscle tissue adaptation in response to endurance training [29–32]. Whether these genes are truly critical for metabolic and performance adaptations to endurance training has yet to be determined. Indeed, training-induced changes in baseline transcriptome have also shown to play an important role [9–11]. Our transcriptomic analysis revealed that the extracellular matrix may be an important factor for skeletal muscle adaptation in response to endurance training. Thus, we argue that mRNA expression changes in human skeletal muscle during later stages of recovery from a single bout of endurance exercise reflect more prolonged molecular responses to short-term energy and ionic homeostasis challenges rather than chronic steady-state adaptation to endurance training [7]. Protein supplementation subtly impacts endurance training-induced changes in the skeletal muscle transcriptome. In addition, our transcriptomic analysis revealed that the extracellular matrix may be an important factor for skeletal muscle adaptation in response to endurance training.

## Methods

### Subjects

**Fig. 4:**
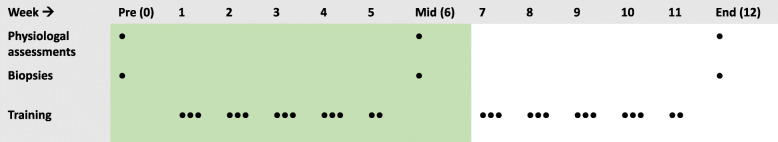
Schematic overview of the study protocol. Forty subjects completed 10 wk. of progressive endurance training while consuming either 25 g carbohydrates or 25 g protein post-exercise and daily before sleep. All measurements were assessed before, midterm (week 6) and after (week 12). Strongest effect of protein supplementation was observed following 5 weeks of endurance training. To gain more insight into mechanisms underlying greater physiological adaptation as a result of protein supplementation we analyzed skeletal muscle transcriptome data from baseline to midterm. Black dots: measurement points, bleu dots: exercise sessions. Grey part: contains physiological and microarray data analyzed for this manuscript

The investigation was approved by the Medical Ethical Committee of Wageningen University, in accordance with the Declaration of Helsinki. This trial was registered at clinicaltrials.gov as NCT03462381 and adheres to CONSORT guidelines for clinical trials. A detailed description of subject participation, experimental design, endurance training program, supplemental strategy, whole-body physiological outcome measures can be found in our previous publication [16]. A schematic overview of the study protocol can be found in Fig. 4.

### Muscle biopsies, sample preparation and microarray analysis

Baseline (week 0) and post-intervention fasted muscle biopsies were taken as described by Bergstrom (1974) [33], and the procedure used can be found elsewhere [16]. Total RNA was isolated from the skeletal muscle tissue by using Trizol reagent (Invitrogen, Breda, Netherlands). Thereafter, RNA was purified using the Qiagen RNeasy Micro kit (Qiagen, Venlo, Netherlands), and RNA quality was checked using an Agilent 2100 bioanalyzer (Agilent Technologies, Amsterdam, Netherlands). Total RNA (100 ng) was labelled using an Affymetrix WT plus reagent kit (Life Technologies, Bleiswijk, Netherlands) and hybridized to human whole genome Genechip Human Gene 2.1 ST arrays, (Life Technologies, Bleiswijk, Netherlands). Sample labelling, hybridization to chips, and image scanning were performed according manufacturer’s instructions.

### Statistics

Statistical analysis of gene expression changes was performed using *limma* R library [34]. Contrasts were set for endurance training effect in both groups and an interaction term was used to determine the effect of protein supplementation (protein group versus the control group). *P*-values were calculated using Intensity Based Moderated t-tests (IBMT) [35]. Significant genes were first selected using the False Discovery Rate Adjusted F-statistic *p*-value < 0.05. Unadjusted *p*-values below 0.01 for the contrasts were considered statistically significant within the genes that passed the F-test. Gene set enrichment analysis was done using pre-ranked lists ranked by the t-values from the *limma* contrasts [36, 37]. We used the most recent library of canonical pathways from The Molecular Signatures Database (MsigDb) [36]. An adjusted p-value (q-value) of 0.10 was considered significant for the gene rest enrichment analysis results. Venn diagram and Heatmaps were made using the ComplexHeatmap library [38] and GraphPad Prism 8.01 for Windows (San Diego, CA). EnrichR was used to determine differences in GO biological processes [39, 40]. A detailed description of the statistical analysis used for the physiological data can be found in our previous publication [16].

## Data Availability

Microarray data will be publicly available at Gene Expressions Omnibus (GEO) repository and as supporting file: GPL28236.

## Abbreviations

AMP-activated protein kinase: Adenosine monophosphate-activated protein kinase
A2M: And alpha-2-macroglobulin
CON: Control group
COL4A1: Collagen type IV alpha 1 chain
COL4A2: Collagen type IV alpha chain
COL3A1: Collagen type III alpha 1 chain
IBMT: Intensity Based Moderated t-tests
LAMA4: Laminin subunit alpha 4
LAMB1: Laminin subunit beta 1
mRNA: Messenger ribonucleic acid
NID1: Nidogen 1
PXDN: Peroxidasin
PRO: Protein group
SPARC: Secreted protein acidic and cysteine rich
MsigDb: The Molecular Signatures Database
